# Reusable Magnetic Nanoparticle Immobilized Nitrogen-Containing Ligand for Classified and Easy Recovery of Heavy Metal Ions

**DOI:** 10.3390/molecules25143204

**Published:** 2020-07-14

**Authors:** Jingyun Jing, Congling Shi

**Affiliations:** Beijing Key Laboratory of Metro Fire and Passenger Transportation Safety, China Academy of Safety Science and Technology, Beijing 100012, China; jingjingyun14@iccas.ac.cn

**Keywords:** magnetic nanoparticle, surface modification, adsorption, heavy metal ions, regeneration

## Abstract

Functionalized Tris[2-(dimethylamino) ethyl] amine (Me_6_TREN) ligands tethered-Fe_3_O_4_@Me_6_TREN nanoparticles (NPs) with a size of 150 nm were prepared to achieve classified and easy recovery of heavy metal ions in wastewater. The preparation of such NPs related to sequential silane ligand exchange and a following cure and Schiff base reactions for Fe_3_O_4_ NPs. Fe_3_O_4_@Me_6_TREN NPs as an effective nano-adsorbent of heavy metals exhibited significant differences in maximum adsorption capacity for Cr(III) (61.4 mg/g), Cu(II) (245.0 mg/g), Pb(II) (5.3 mg/g), and Cd(II) (1136.2 mg/g), in favor of classified removal of heavy metals from wastewater. Furthermore, Fe_3_O_4_@Me_6_TREN NPs can be regenerated by desorbing metal ions from NP surfaces eluted with ethylenediaminetetraacetic acid disodium salt (EDTA-Na_2_) aqueous, which endows such NPs promising potency as new nano-vectors for the removal of heavy metals.

## 1. Introduction

Increasingly severe water pollution caused by heavy metals has posed a serious threat to human health [1], which is in a situation of urgent need to be handled worldwide [2,3,4]. Electroplating wastewater as the main pollution source is hazardous to the human body with a variety of heavy metal ions exceeding the standard [5,6,7]. The maximal permissible limit of Cr(III), Cu(II), Pb(II), and Cd(II) ions in drinking water is 0.05 mg/L, 2 mg/L, 0.01 mg/L, and 0.003 mg/L, respectively based on the World Health Organization [8].

Various efforts, such as chemical precipitation [9,10], ion exchange [11,12], membrane filtration [13,14], and adsorption [15,16] have been taken to remove heavy metal ions from wastewater. Among them, the adsorption method with activated carbon [17,18], carbon nanotube [19], sewage sludge ash [20], chitosan [21,22], and hydrogel [23] as adsorbents has been recognized as the most promising candidate for removal of heavy metals from polluted water [24,25] with superiority in cost and efficiency. Further, magnetic composite nanoparticles which can be removed conveniently from water with the help of an external magnet because of their exceptional properties possess better performance in removing heavy metal ions from water [26,27]. However, selective recovery of metal ions from water are challenging, which limits wide application of this method. Therefore, the development of new magnetic nano-adsorbents [28] with simplicity, effectiveness, and recyclability has great significance in classified recovery of heavy metal ions in wastewater.

**Scheme 1:**
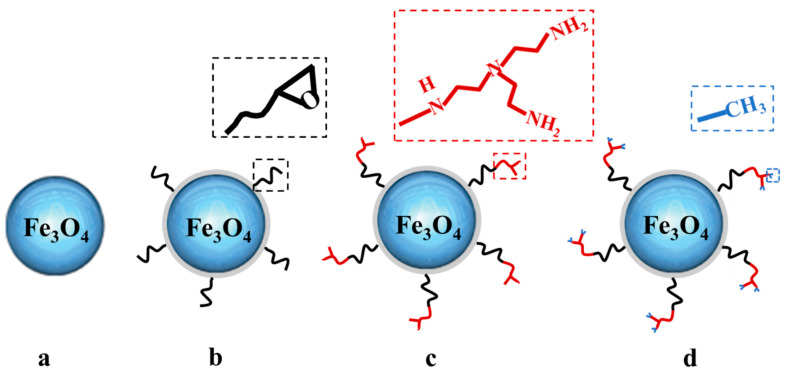
Synthesis of Fe_3_O_4_@Me_6_TREN nanoparticles (NPs): (**a**) Fe_3_O_4_ NP; (**b**) Fe_3_O_4_@epoxide NP; (**c**) Fe_3_O_4_@TAEA NP; (**d**) Fe_3_O_4_@Me_6_TREA NP.

Here, we aim to propose a facile approach to graft Me_6_TREN ligands onto the surface of SiO_2_-coated Fe_3_O_4_ to prepare functionalized Fe_3_O_4_@Me_6_TREN nanoparticles (NPs) to act as a new nano-adsorbent of heavy metals in polluted water. As shown in Scheme 1, based on magnetic polyethylene glycol (PEG)-capped Fe_3_O_4_ NPs synthesized via solvothermal method, silane ligand exchange and a following cure, Schiff base reactions and reduction by NaBH_4_ were sequentially performed to make Me_6_TREN-alike ligands tethered onto such NPs. Fe_3_O_4_@Me_6_TREN NPs as a powerful nano-gripper can selectively concentrate some heavy metals to realize classified and easy recovery. Furthermore, the NPs can be regenerated by washing with stronger chelators of heavy metal ions, such as ethylenediaminetetraacetic acid disodium salt (EDTA-Na_2_) aqueous. Such NPs with magnetic responsiveness and recyclability are a competitive medium for complexation of heavy metals.

## 2. Results and Discussion

### 2.1. The Fe_3_O_4_@epoxide NPs

**Figure 1:**
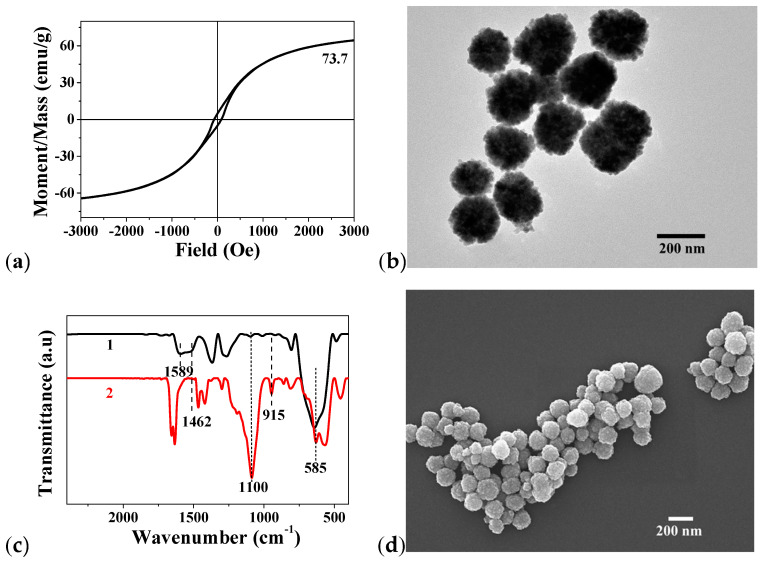
(**a**) VSM curve of the Fe_3_O_4_ NP; (**b**) TEM image of Fe_3_O_4_ NP; (**c**) Fourier transform infrared (FT-IR) spectra of Fe_3_O_4_ NP (1) and Fe_3_O_4_@epoxide NP (2); (**d**) SEM image of Fe_3_O_4_@epoxide NP.

The Fe_3_O_4_ NPs were synthesized by the solvothermal method as reported previously [29]. The crystalline structure of the obtained NPs was confirmed by the XRD spectrum (Figure S1). The Fe_3_O_4_ NPs are ferromagnetic with a saturation magnetization of 73.7 emu/g and coercivity at 86 Oe (Figure 1a). The NPs were well-dispersed in ethanol with an approximate diameter of 150 nm (Figure 1b). The epoxide groups were then introduced onto the NP surface by sol-gel coating of an epoxide-functional silane KH560 [30]. The unmodified NPs show an absorption peak at 1462 cm^−1^, which is assigned to the -CH_2_- group of PEG. The strong IR band at 585 cm^−1^ is characteristic of the Fe-O vibrations related to the ferrite core. After KH560 treatment, the IR spectrum of the NPs displayed a strong absorption peak at 1100 cm^−1^, which is characteristic of the Si-O-Si vibrations, indicating the formation of a silane layer on the NP surface. A peak at 915 cm^−1^ is evidence of the presence of epoxide group [31] (Figure 1c). Meanwhile, the modified NPs retain their original spherical shape and size as shown in the SEM image (Figure 1d).

### 2.2. The Fe_3_O_4_@Me_6_TREN NP

**Figure 2:**
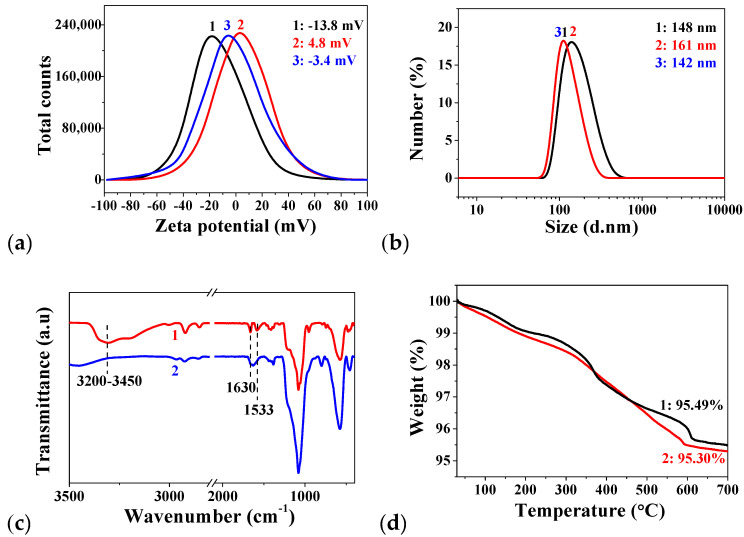
(**a**) Zeta potential and (**b**) DLS traces of Fe_3_O_4_@epoxide NP (1), Fe_3_O_4_@TAEA NP (2), and Fe_3_O_4_@Me_6_TREN NP (3); (**c**) FT-IR spectra of Fe_3_O_4_@TAEA NP (1) and Fe_3_O_4_@Me_6_TREN NP (2); (**d**) thermogravimetric analysis (TGA) curves of Fe_3_O_4_@epoxide NP (1) and Fe_3_O_4_@ Me_6_TREN NP (2).

The Fe_3_O_4_@TAEA NPs were synthesized by cure reaction of amino and epoxide groups with treatment of excessive TAEA against Fe_3_O_4_@epoxide NPs. To avoid crosslinking, 10-fold excess of TAEA was added, so only one out of three amino groups reacted with the epoxide. With the amino groups facing outward, zeta potential of the NPs dispersed in ethanol undergoes an obvious transformation from –13.8 mV (Figure 2a, curve 1) to 4.8 mV (Figure 2a, curve 2) after TAEA grafting. The two unreacted amino groups per TAEA unit were further reacted with formaldehyde under acidic conditions to form a Schiff base, and then reduced by NaBH_4_. When all the primary amine groups were converted to tertiary amine, the Fe_3_O_4_@Me_6_TREN NPs dispersed in ethanol holding a zeta potential of –3.4 mV (Figure 2a, curve 3) and a diameter of 142 nm (Figure 2b, curve 3) were obtained. In the IR spectra, the peaks assigned to -NH_2_ group in primary amine between 3200–3450, 1533 and 1630 cm^−1^ completely disappeared after formation of tertiary amine (Figure 2c), which is evidence of successful surface modification. TGA measurements were performed to estimate the exact content of Me_6_TREN ligands on the NP surface. The weight loss of the Fe_3_O_4_@Me_6_TREN NPs increased by 0.19% compared to that of Fe_3_O_4_@epoxide NPs after heating to 700 °C, indicating that the NPs contain 0.20 wt% of Me_6_TREN functionalities (Figure 2d).

### 2.3. Adsorption of Heavy Metal Ions in Water with Fe_3_O_4_@Me_6_TREN NPs

**Figure 3:**
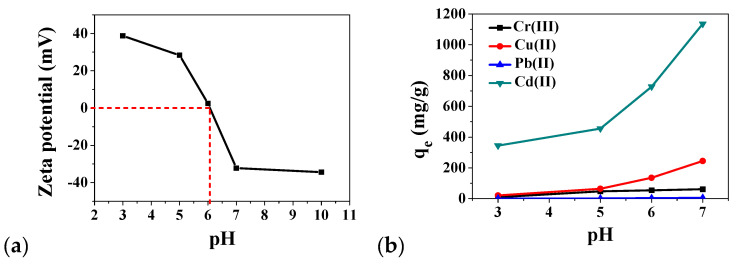
(**a**) Zeta potential of Fe_3_O_4_@Me_6_TREN NPs aqueous dispersion at various pH values; (**b**) the effect of pH on adsorption capacity for four metal ions by Fe_3_O_4_@Me_6_TREN NPs.

The Fe_3_O_4_@Me_6_TREN NPs can serve as nano-grippers of heavy metal ions by complexation to achieve removal of metal ions from water. The zeta potential of aqueous solution of Fe_3_O_4_@Me_6_TREN NPs undergoes a positive-to-negative transformation with the increase of pH value in the range from 3 to 7 and the isoelectric point falls around 6.1 measured on Nano ZS (Figure 3a), which has an influence on the adsorption performance for metal ions. The effect of pH value on adsorption capacity for four heavy metal ions (Cr(III), Cu(II), Pb(II), and Cd(II)) was studied by a UV–Vis spectrophotometer with an initial concentration of ~5 mg/mL for each metal salt compound. Standard curves of UV–Vis adsorption of four metal ions in water were measured beforehand (Figures S2–S5). Saturated adsorption capacity for metal ions can be calculated based on the Formula (1)

(1) $$q_{e}=\frac{\left(C_{0}-C_{e}\right)\times V}{m}$$

where *q*_e_ is equilibrium adsorption capacity; *C*_0_ and *C*_e_ refer to the initial and equilibrium concentrations of metal ions in solution, respectively; *V* is the volume of solution; *m* stands for the mass of adsorbent. As shown in Figure 3b, the adsorption capacity of all four metal ions by Fe_3_O_4_@Me_6_TREN NPs increases in the solution pH value window of 3–7 for weaker electrostatic repulsion between NP surfaces and metal ions in a higher pH value. However, the adsorption capacity of the NPs for four metal ions exhibits a great difference, which is the strongest for Cr(III) (61.4 mg/g), Cu(II) (245.0 mg/g), and Cd(II) (1136.2 mg/g), and the weakest for the Pb(II) ion (5.3 mg/g). The significant difference of adsorption capacity of the Fe_3_O_4_@Me_6_TREN NPs for the selected four metal ions is beneficial for the classified recovery of heavy metal ions in wastewater.

**Figure 4:**
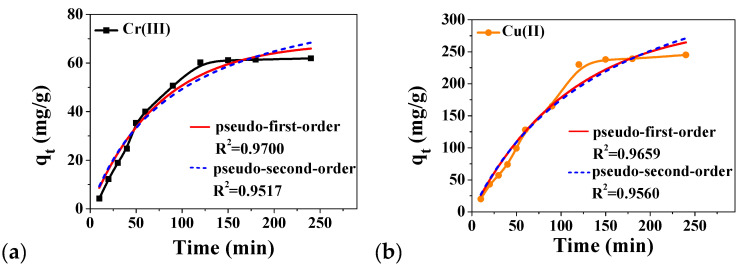

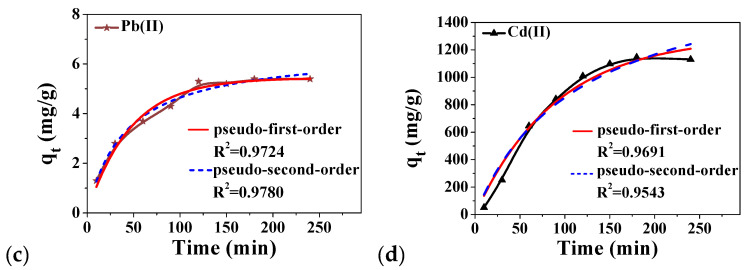
(**a**–**d**) Adsorption kinetic curves of four metal ions by Fe_3_O_4_@Me_6_TREN NPs: (**a**) Cr(III); (**b**) Cu(II); (**c**) Pb(II); (**d**) Cd(II).

Similar batch experiments were performed to determine the effect of contact time on adsorption capacity. As Figure 4a–d illustrates, although adsorption capacity of Fe_3_O_4_@Me_6_TREN NPs for four metal ions shows a dramatic difference, the dynamic adsorption process experiences a similar trend in a rapid growth in the early stage (50 min), followed by a gradual equilibrium process after approximately 120 min. Furthermore, to reveal the adsorption behavior of the NPs for Cr(III), Cu(II), Pb(II), and Cd(II) ions, adsorption kinetic data were fitted with two common models of pseudo-first-order Formula (2) and pseudo-second-order Formula (3) [32] (Figure 4a–d).

(2) $$\mathrm{Pseudo-first-order}:\;q=q_{e}\left(1-e^{-k_{1}t}\right)$$

(3) $$\mathrm{Pseudo-second-order}:\;q=\frac{{q_{e}}^{2}k_{2}t}{1+q_{e}k_{2}t}$$

Details of kinetic parameters fitted with the two models are provided in Table 1. According to the results, the nonlinear fitting curve of the pseudo-first-order model presents a better correlation for Cr(III), Cu(II), and Cd(II) ions; the correlation coefficient R^2^ values are all higher than 0.96, which is slightly larger than that of the pseudo-second-order model. The behavior of adsorption of Pb(II) ion supporting a pseudo-second order equation is in agreement with chemisorption being the rate controlling step [33].

**Figure 5:**
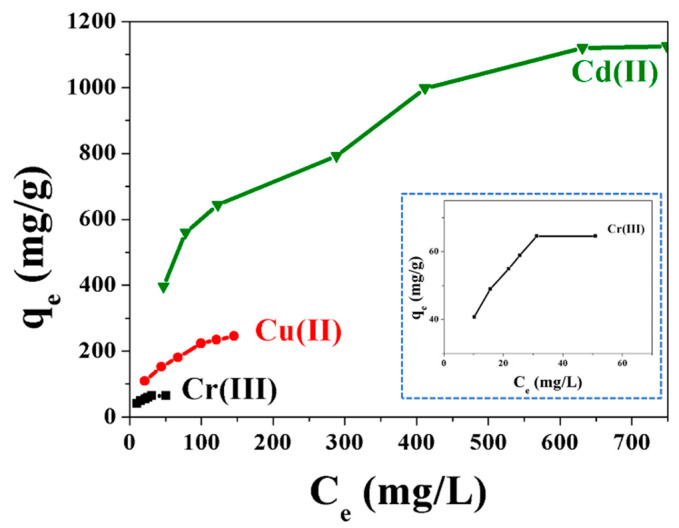
Adsorption isotherms of Fe_3_O_4_@Me_6_TREN NPs for Cr(III), Cu(II), and Cb(II) ions.

The adsorption isotherms of magnetic NPs for Cr(III), Cu(II), and Cd(II) ions are presented in Figure 5. The Langmuir and Freundlich models were used to simulate the adsorption isotherms, which can be represented in the following Equations (4) and (5):

(4) $$\mathrm{Langmuir}\;\mathrm{isotherm}:\;\frac{C_{e}}{q_{e}}=\frac{C_{e}}{q_{m}}+\frac{1}{K_{L}q_{m}}$$

(5) $$\mathrm{Freundlich}\;\mathrm{isotherm}:\;lgq_{e}=lgK_{F}+\frac{1}{n}lgC_{e}$$

where *C_e_* (mg/L) is the equilibrium concentration of metal ions in solution; *q_e_* (mg/g) and *q_m_* (mg/g) are the equilibrium and maximum adsorption capacity, respectively; *K_L_* (L/mg) is the constant of Langmuir isotherm; *K_F_* and n are the constants of the Freundlich isotherm.

The parameters related to the Langmuir and Freundlich isotherm models were given in Table 2. According to the results, the Langmuir model represents a little more goodness-of-fit than the Freundlich model for three metal ions based on the correlation coefficient (*R*^2^). Furthermore, 1/n as the heterogeneity factor to describe the favoring degree of adsorption process was less than 0.5, suggesting superbly favorable adsorption for the three metal ions.

### 2.4. Desorption of Heavy Metal Ions from Fe_3_O_4_@Me_6_TREN NPs

**Figure 6:**
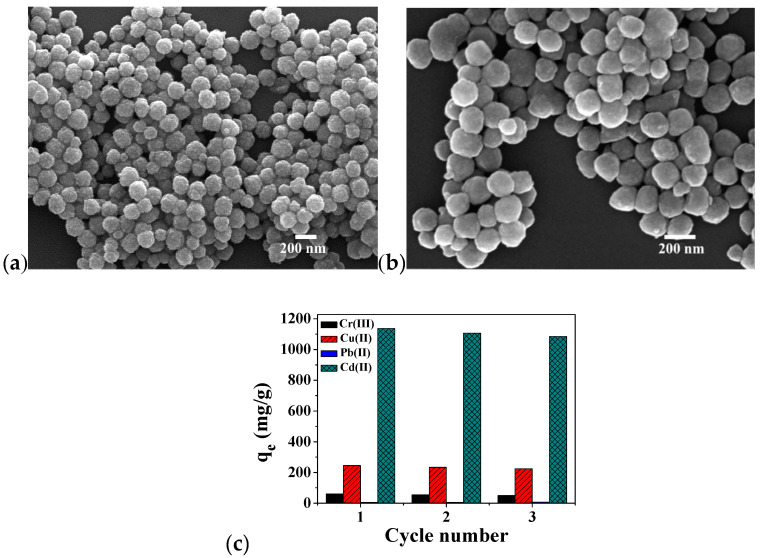
SEM images of Fe_3_O_4_@Me_6_TREN NPs after (**a**) adsorption and (**b**) desorption of heavy metal ions, respectively; (**c**) adsorption capacity of NPs after recycling three times.

The Fe_3_O_4_@Me_6_TREN NPs can be regenerated by desorbing metal ions from NP surfaces with EDTA-Na_2_ (1:1 to the mol of metal ions) as a stronger chelator. To study the desorption efficiency, a comparison between adsorption mass (*m_1_*) and desorption mass (*m_2_*) per mg of NPs for each metal ion was analyzed using the concentration of the residual metal ion in solution after adsorption (*C_1_*) and that after desorption (*C_2_*) measured by ICP-MS. As shown in Table 3, EDTA-Na_2_ exhibited excellent adsorption capacity for these metal ions, thus causing a desorption efficiency higher than 95% for metal ions except the Pb(II) ion from NPs. Furthermore, there was no significant difference in the shape and size of Fe_3_O_4_@Me_6_TREN NPs after recycling, observed in SEM (Figure S6), and the element composition of NPs before and after desorption were characterized by SEM-EDX (Figure S6). In addition, NPs possessed a steady adsorption capacity for metal ions after three cycles (Figure 6c), meaning that such NPs are of great potentiality for reuse.

## 3. Materials and Methods

### 3.1. Materials

Ferric chloride hexahydrate (FeCl_3_·6H_2_O), ethylene glycol, ethylenediaminetetraacetic acid disodium salt (EDTA-Na_2_), formaldehyde solution (38% in water), sodium acetate, anhydrous cupric sulfate (CuSO_4_), lead denitrate (Pb(NO_3_)_2_), cadmium nitrate (Cd(NO_3_)_2_), and chromic chloride hexahydrate (CrCl_3_·6H_2_O) were purchased from Sinopharm Chemical Reagent. 3-Glycidoxypropyltrimethoxy silane (KH560) and tris(2-aminoethyl) amine (99%) were purchased from J&K Scientific. Polyethylene glycol (PEG, Mn = 20 kg/mol) was purchased from Sigma Aldrich. Sodium borohydride (NaBH_4_) was purchased from Jinke Institute of Fine Chemicals. Acetic acid, hydrochloric acid, acetonitrile, ethylene glycol, and ethanol absolute (analytical reagent, AR) were purchased from Beijing Chemical Reagent. Toluene was purchased from XiLong Scientific. All reagents were used as received unless specified.

### 3.2. Synthesis of PEG-Capped Fe_3_O_4_ NPs

FeCl_3_·6H_2_O (2.36 g, 8.74 mmol), PEG (1.75 g, 8.75 × 10^−2^ mmol), and sodium acetate (3.14 g, 38 mmol) were dissolved in 70 mL of ethylene glycol under sonication. The yellow viscous liquid was then transferred into a sealed stainless steel autoclave with PTFE lining and heated at 180 °C for 8 h. After the autoclave was cooled down, the product NP was collected with a laboratory neodymium magnet, washed with water and ethanol three times, and stored in ethanol to avoid aggregation.

### 3.3. Synthesis of Epoxide-Capped Fe_3_O_4_ NPs (Fe_3_O_4_@epoxide)

PEG-capped Fe_3_O_4_ NPs were pre-treated by sonication in 0.1 M HCl for 10 min to generate more hydroxyl groups on the NP surface. A dispersion was prepared by adding 0.30 g of pre-treated Fe_3_O_4_ NPs into 100 mL of ethanol containing 3.0 μL of acetic acid. Then, 0.10 g (0.42 mmol) KH560 was added dropwise, and the mixture was stirred for 12 h at room temperature. The Fe_3_O_4_@epoxide NPs were washed with ethanol, collected with a magnet, and redispersed in ethanol for storage.

### 3.4. Synthesis of Tris(2-aminoethyl) Amine-Grafted Fe_3_O_4_ NPs (Fe_3_O_4_@TAEA)

To a dispersion of 0.1 g Fe_3_O_4_@epoxide NPs in 50 mL ethanol, 6.3 mL (10-fold excessive of theoretical epoxide content) of tris(2-aminoethyl) amine were added. The reaction mixture was stirred for 12 h at 60 °C to ensure a full reaction between the epoxy and amino groups. The obtained Fe_3_O_4_@TAEA NPs were then collected with a magnet, washed, and redispersed in ethanol.

### 3.5. Synthesis of Me_6_TREN-Grafted Fe_3_O_4_ NP Ligand (Fe_3_O_4_@Me_6_TREN)

To a dispersion containing 0.1 g Fe_3_O_4_@TAEA NPs and 60 μL of acetic acid in 50 mL acetonitrile, 0.5 mL of 38% formaldehyde solution was added. The reactants were stirred at room temperature for 2 h. Subsequently, the dispersion was cooled down to 0 °C, and a solution of 0.10 g NaBH_4_ in 0.5 mL water was slowly added within 30 min via a syringe pump. Then the temperature was raised to 35 °C and kept for 24 h. The resulting Fe_3_O_4_@Me_6_TREN NPs were washed with ethanol, collected with a magnet, and then dried in a vacuum oven.

### 3.6. Adsorption of Heavy Metal Ions in Water with Fe_3_O_4_@Me_6_TREN NPs

Next, 2 mg of Fe_3_O_4_@Me_6_TREN NPs were dispersed in 6 mL of Cr(III), Cu(II), Pb(II), and Cd(II) ions aqueous with an initial concentration (C_0_) of 5 mg/mL for each metal salt compound and different pH value at 30 °C to investigate the effect of pH value on adsorption capacity. Similar experiments of adsorption kinetics were performed at C_0_ = 5 mg/mL, pH = 7, and 30 °C. Adsorption isotherms were investigated with the initial concentration of metal ions ranging from 10 to 800 mg/L at pH = 7. The concentration of metal ions in water was measured by a UV–Vis spectrophotometer.

### 3.7. Desorption of Heavy Metal Ions from Fe_3_O_4_@Me_6_TREN NPs

EDTA-Na_2_ (same equiv. as metal ions) was dissolved into 3 mL aqueous dispersion containing Fe_3_O_4_@Me_6_TREN/metal ion NPs at pH = 4. The mixture was stirred at 30 °C for 2 h. Fe_3_O_4_@Me_6_TREN NPs were recycled by a magnet. The remaining supernatant was diluted with water and then determined by inductively coupled plasma mass spectrometry (ICP-MS) to detect the metal ions content.

### 3.8. Characterization

Transmission electron microscopy (TEM) measurements were performed on JEM-1011 at an accelerating voltage of 100 kV. Scanning electron microscopy (SEM) measurements were performed on Hitachi S-4800 at an accelerating voltage of 15 kV. Particle size distribution and zeta potential were measured on Nano ZS (Malvern Instruments). Crystallinity of the NPs was characterized by Rigaku D/max-2500. The magnetic property was measured using PPMS-9 (Quantum Design Inc., USA) at a magnetic field of 1 T. Fourier transform infrared (FT-IR) spectra were collected on a Bruker Equinox 55 spectrometer. Thermogravimetric analysis (TGA) was performed on PerkinElmer Pyris 1 in air at a heating rate of 10 °C/min. UV–Vis spectrophotometry was performed on TU-1901 to measure the concentration of four heavy metal ions in water. Inductively coupled plasma mass spectrometry (ICP-MS) measurement was performed on Thermo iCAP RQ to analyze the content of the metal ions.

## 4. Conclusions

Versatile and recyclable Fe_3_O_4_@Me_6_TREN NPs as an efficient nano-adsorbent for the removal of heavy metal ions in wastewater were successfully prepared by a simple method in this work. The coating Me_6_TREN is responsible for immobilization of heavy metal ions on NP surfaces with selective adsorption efficiency while the core Fe_3_O_4_ is in charge of magnetic responsiveness. Furthermore, Fe_3_O_4_@Me_6_TREN NPs can be regenerated by desorbing heavy metal ions from NPs with EDTA-Na_2_, which will be of great significance for cost reduction and further industrial application. It endows the Fe_3_O_4_@Me_6_TREN NPs with the ability to be a powerful nano-catcher for classified recovery of heavy metal ions, which can be manipulated by the magnetic field.

## Figures and Tables

**Table 1:** Adsorption kinetic parameters of the Fe_3_O_4_@Me_6_TREN NPs for Cr(III), Cu(II), Pb(II), and Cd(II) ions.

Metal Ions	Pseudo-First-Order			Pseudo-Second-Order		
	*q_e_* (mg·g^−1^)	*k*_1_ (min^−1^)	*R* ^2^	*q_e_* (mg·g^−1^)	*k*_2_ (mg·g^−1^·min^−1^)	*R* ^2^
Cr(III)	68.7	1.34 × 10^−2^	0.9700	94.5	2.20 × 10^−5^	0.9517
Cu(II)	298.0	0.91 × 10^−2^	0.9659	446.2	1.47 × 10^−5^	0.9556
Pb(II)	5.4	2.14 × 10^−2^	0.9724	6.6	3.60 × 10^−3^	0.9780
Cd(II)	1298.0	1.11 × 10^−2^	0.9691	1842.8	4.67 × 10^−6^	0.9543

**Table 2:** Adsorption isotherm parameters for the removal of Cr(III), Cu(II), and Cd(II) ions by the Fe_3_O_4_@Me_6_TREN NPs.

Metal Ions	Langmuir			Freundlich		
	*q_m_*(mg/g)	*K_L_* (L/g)	*R* ^2^	*K_F_*	1/n	*R* ^2^
Cr(III)	76.6	0.122	0.989	15.85	0.404	0.987
Cu(II)	323.6	0.023	0.990	33.11	0.411	0.983
Cd(II)	1312.3	0.008	0.987	100.00	0.376	0.975

**Table 3:** Inductively coupled plasma mass spectrometry (ICP-MS) results of adsorption and desorption of various heavy metal ions.

Metal Ions	Adsorption		Desorption	
	*C_1_* (ng/g)	*m_1_* (mg)	*C_2_* (ng/g)	*m_2_* (mg)
Cr(III)	157.52	0.059	44.60	0.061
Cu(II)	208.51	0.254	127.56	0.242
Pb(II)	775.97	0.004	6.29	0.003
Cd(II)	236.23	1.003	435.71	0.956

## Supplementary Materials

The following are available online, Additional XRD pattern, standard UV–Vis curves, and SEM-EDX images are included.
