# Supplementary material for: Reusable Magnetic Nanoparticle Immobilized Nitrogen-Containing Ligand for Classified and Easy Recovery of Heavy Metal Ions
Source: Molecules. 2020 Jul 14;25(14):3204. doi: 10.3390/molecules25143204 (PMC7397339; doi:10.3390/molecules25143204)
Supplement: Supplementary file 1 [file molecules-25-03204-s001.pdf]

Reusable Magnetic Nanoparticle Immobilized Nitrogen-Containing Ligand for Classified and Easy Recovery of Heavy Metal Ions

Jingyun Jing and Congling Shi*

Beijing Key Laboratory of Metro Fire and Passenger Transportation Safety, China Academy of Safety Science and Technology, Beijing 100012, China

* Corresponding author.

E-mail: shicl@chinasafety.ac.cn

Postal address: yard 32, Beiyuan Road, Chaoyang District, Beijing, China

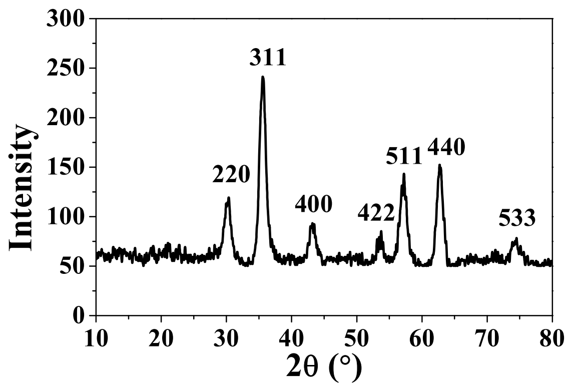

Figure S1. XRD pattern of prepared the Fe_3O_4 NP.

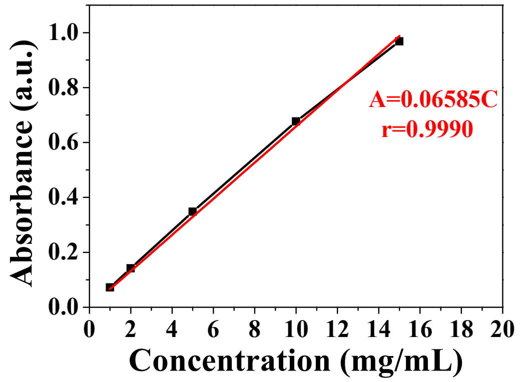

Figure S2. Standard curve of UV-Vis adsorption of $CrCl_3 \cdot 6H_2O$ in water.

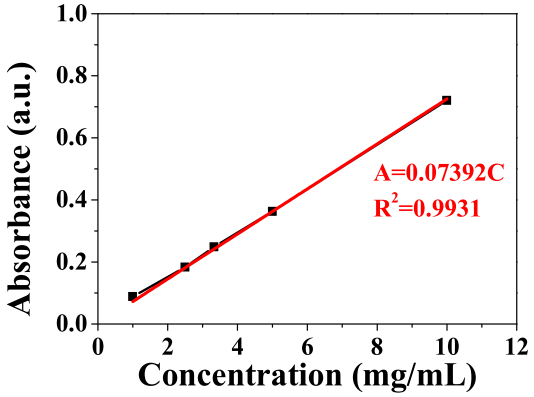

Figure S3. Standard curve of UV-Vis adsorption of $CuSO_4$ in water.

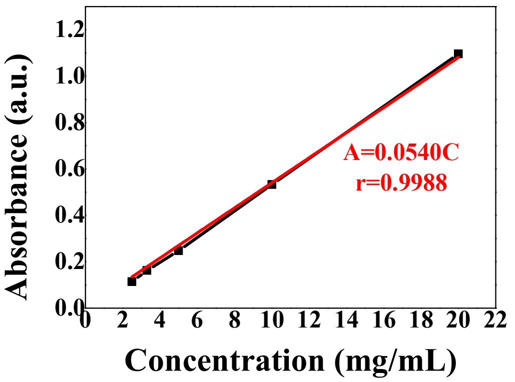

Figure S4. Standard curve of UV-Vis adsorption of $Pb(NO_3)_2$ in water.

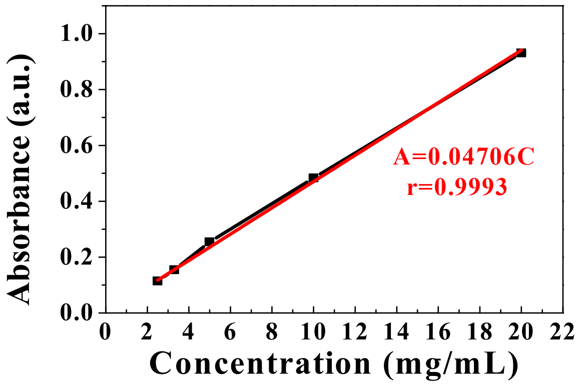

Figure S5. Standard curve of UV-Vis adsorption of $Cd(NO_3)_2$ in water.

a)

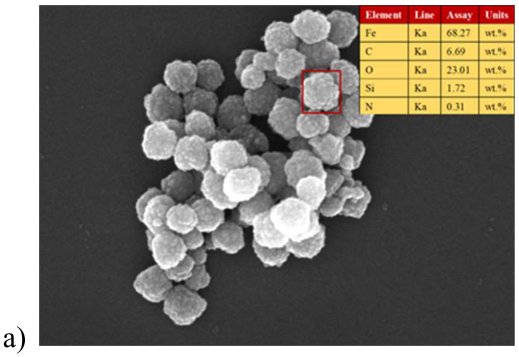

b)

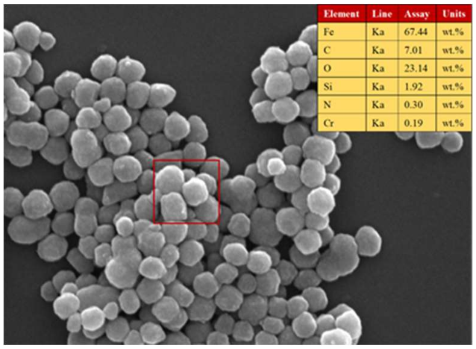

c)

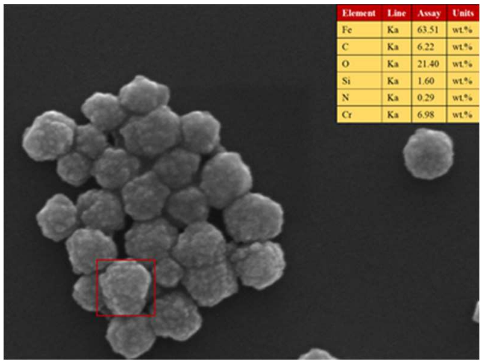

Figure S6. a-c) refers to EDX analysis of Fe_3O_4@Me_6TREN NPs, Fe_3O_4@Me_6TREN NPs with adsorption of Cr(III) and Fe_3O_4@Me_6TREN NPs after desorption of Cr(III) respectively.
